# Compensatory evolution facilitates loss of *prfB* autoregulation in *Pseudomonas fluorescens* SBW25

**DOI:** 10.1093/molbev/msag125

**Published:** 2026-05-26

**Authors:** Sungbin Lim, Frederic Bertels, Javier Lopez-Garrido, Jenna Gallie

**Affiliations:** Max Planck Institute for Evolutionary Biology, Plön 24306, Germany; Max Planck Institute for Evolutionary Biology, Plön 24306, Germany; Max Planck Institute for Evolutionary Biology, Plön 24306, Germany

**Keywords:** compensatory evolution, translation, programmed ribosomal frameshifting, release factor 2, *prfB, Pseudomonas fluorescens*

## Abstract

Understanding why some traits are maintained whereas others are repeatedly lost is a central question in evolutionary biology. Here, we address this question through the evolutionary dynamics of autoregulation of *prfB*, which encodes peptide-chain release factor 2 (RF2), a factor in bacterial translation termination. RF2 recognizes UGA and UAA stop codons and catalyzes polypeptide release. In many species, *prfB* contains an internal UGA stop codon that causes premature termination by RF2. Full RF2 synthesis depends on a + 1 programed ribosomal frameshifting (PRF) event at this stop codon, which occurs more frequently when RF2 levels are low, resulting in autoregulation of *prfB* expression. While widespread, this mechanism has been lost repeatedly across bacteria. We combined phylogenetics, experimental evolution, and molecular genetics to investigate the evolutionary forces underlying this loss. Phylogenetically informed analyses revealed no significant correlation between autoregulation and UGA stop codon usage, and autoregulation elimination in *Pseudomonas fluorescens* SBW25 had no detectable fitness effect. However, engineered mutations that reduced frameshifting at the *prfB* autoregulatory site caused fitness defects that were compensated by two classes of mutation: mutations affecting ribosome-associated proteins (RsmA, RsmH, RplI), and single-nucleotide deletions in *prfB* that adjusted the reading frame to bypass the internal stop codon, eliminating autoregulation. These results suggest that loss of *prfB* autoregulation can be facilitated by compensatory mutations when frameshifting at the *prfB* autoregulatory site is compromised and RF2 production is insufficient. Our findings illustrate how compensatory evolution can favor trait loss when the fitness benefit of losing the trait outweighs its cost.

## Introduction

Translation fidelity is essential for cellular homeostasis, and a critical step is the accurate recognition of stop codons during termination. This process relies on ribosome release factors (RFs), which bind to stop codons and catalyze the hydrolysis of the peptide bond between the nascent polypeptide and the tRNA in the peptidyl (P) site, thereby releasing the complete protein and promoting ribosome disassembly. While a single RF recognizes all three stop codons in eukaryotic cells, bacteria possess two RFs: RF1, which recognizes UAG and UAA, and RF2, which recognizes UGA and UAA.

**Figure 1:**
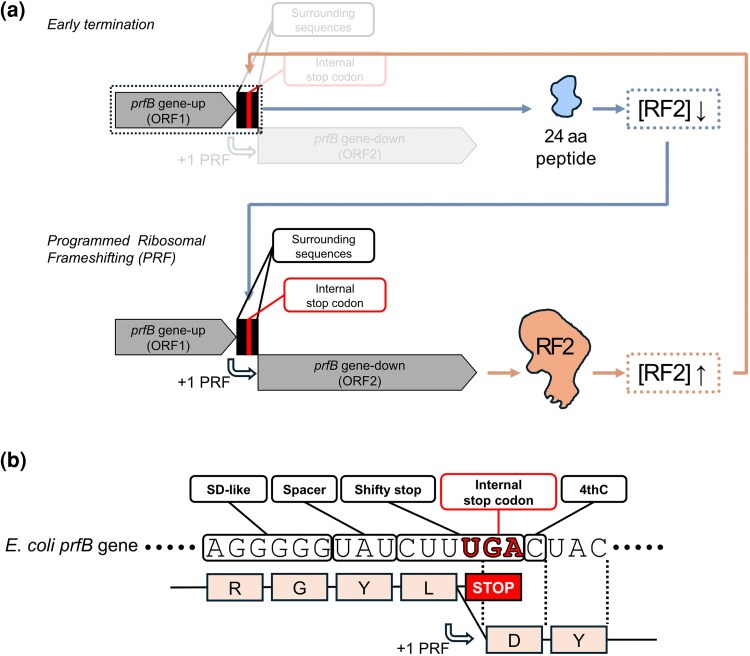
Programed ribosomal frameshifting (PRF) in *prfB*. a) Diagram of *prfB* autoregulation. The *prfB* locus is depicted as two gene arrows—*prfB* gene-up (ORF1) and *prfB* gene-down (ORF2)—separated by an internal UGA stop codon. Black boxes flanking the UGA indicate sequences required for frameshifting (see panel B for details). Synthesis of full-length RF2 requires a + 1 programed ribosomal frameshift (+1 PRF) that bypasses termination at the internal UGA. When RF2 concentration is high (upper panel, early termination), the internal UGA is efficiently recognized, causing premature termination and production of an unstable 24-aa peptide. When RF2 concentration is low (lower panel, PRF), the +1 frameshift occurs at high frequency, bypassing the internal stop and yielding full-length RF2, thereby increasing RF2 levels. This autoregulatory loop maintains RF2 homeostasis. The orange line indicates the functional path in which increased frameshifting raises RF2 concentration; the blue line shows the opposite scenario. b) Diagram showing functionally important elements in the PRF site of *E. coli prfB* gene. The UGA stop codon is in red. Additional elements that promote frameshifting are indicated: a Shine-Dalgarno (SD)-like sequence (AGGGGG), a UAU spacer codon, the shifty-stop sequence (CUU UGA) and the C at the nucleotide immediately following the UGA stop codon (4thC). The amino acids produced by translation before and after the +1 frameshift (+1 PRF) are indicated below the RNA sequence. See main text for details.

Although precise recognition of stop codons by RFs is critical for translation fidelity, not all stop codons are equally efficient (Adamski et al. 1993; Poole et al. 1995; Pavlov et al. 1998; Higashi et al. 2006; Betney et al. 2010; Wei and Xia 2017; Zhang et al. 2020; Biziaev et al. 2022; Riegger and Caliskan 2022; Loughran et al. 2023). Some are prone to be bypassed at high rates due to ribosomal frameshifting (Brown et al. 1993, 1994), and in certain cases these inefficient stop codons have been co-opted for gene regulation or the production of alternative protein isoforms (Wong et al. 2008; Antonov et al. 2013; Caliskan et al. 2017; Fan et al. 2017). A well-documented example is the autoregulation of *prfB*, the gene encoding RF2, which contains a highly inefficient UGA stop codon within its reading frame (Craigen et al. 1985; Craigen and Caskey 1986; Weiss et al. 1988) (Fig. 1a). Recognition of the internal stop by RF2 results in translation termination after only 24 residues in *Escherichia coli*, yielding an unstable peptide that is rapidly degraded (Williams et al. 1989). Alternatively, the internal stop can be bypassed by a + 1 programed ribosomal frameshift (PRF), allowing the synthesis of full-length RF2 (Donly et al. 1990; Mikuni et al. 1991). The balance between termination and frameshifting is affected by RF2 levels: low levels increase frameshifting frequency and the production of full-length RF2, whereas high levels promote termination, creating a negative feedback loop (Fig. 1a).

The mechanics of the PRF event have been best characterized in *E. coli*. The PRF site spans 16 nucleotides and includes several elements that influence frameshifting efficiency (Fig. 1b) (throughout the manuscript, nucleotide annotations at PRF site are given in RNA notation, using U instead of T). The most critical component is the “shifty stop” sequence (CUU UGA), which favors +1 frameshifting via wobble base pairing between tRNA-Leu(GAG) and the mRNA (Weiss et al. 1987, 1988; Curran and Yarus 1988). Briefly, the CUU codon is decoded by wobble tRNA-Leu(GAG); if the anticodon shifts +1, it can still pair with the new codon UUU. This shift is enhanced by a nearby Shine-Dalgarno (SD)-like sequence (AGG GGG), which transiently stalls the ribosome when the CUU codon is in the P-site, thereby facilitating frameshifting (Weiss et al. 1987, 1988). The spacer codon immediately preceding the “shifty stop” sequence, typically UAU or UCU (Baranov et al. 2002a), also promotes frameshifting by favoring rapid tRNA ejection (Baranov et al. 2004; Devaraj et al. 2009). tRNAs with stronger base pairings slow ejection, and reduce frameshifting (Márquez et al. 2004; Sanders and Curran 2007). Finally, the nucleotide immediately after the stop codon—hereafter referred to as the fourth position—influences termination efficiency, with cytosine particularly enhancing frameshifting (Poole et al. 1995). Together, these features create a tunable regulatory site in which translation termination competes with +1 frameshifting to control RF2 synthesis.

Nearly all naturally occurring bacteria encode RF2, and at least two-thirds have an internal stop codon in *prfB* (Persson and Atkins 1998; Baranov et al. 2002a; Bekaert et al. 2006; Naeem et al. 2023). The PRF-associated elements tend to be highly conserved across species (Baranov et al. 2002a, 2002b; Prince et al. 2025), and rare variability in this region has been correlated with changes in the frameshifting rates (Craigen and Caskey 1986; Weiss et al. 1988; Naeem et al. 2023). Despite this widespread conservation, the physiological relevance of *prfB* autoregulation remains unclear. Autoregulation is often proposed to prevent RF2 overproduction (Betney et al. 2010; de Silva et al. 2010), which may help minimize mis-termination at sense codons similar to UGA (Freistroffer et al. 2000; Prince et al. 2025), such as the tryptophan codon UGG (Abdalaal et al. 2020), or to regulate the frequency of ribosome drop-off during entry to stationary phase (Betney et al. 2010). However, studies in different species have found that deletion of the internal stop has subtle or negligible deleterious effects under laboratory conditions (Johnson et al. 2011; Abdalaal et al. 2020; McNutt et al. 2021; Naeem et al. 2023), and only *prfB* overexpression from inducible promoters have been associated with growth defects (Jørgensen et al. 1993; Rengby and Arnér 2007; Lalanne et al. 2021; Prince et al. 2025). In addition, published surveys indicate that autoregulation is completely absent from as many as one third of sequenced genomes (Persson and Atkins 1998; Baranov et al. 2002a; Prince et al. 2025), suggesting that the mechanism can be lost without fatal fitness consequences in nature.

Here, we have explored the evolutionary forces behind *prfB* autoregulation loss using *P. fluorescens* SBW25 as a model system. As in other species, elimination of *prfB* autoregulation had no detectable fitness effect under standard laboratory conditions. However, mutations in the PRF site that reduced frameshifting efficiency led to severe fitness defects. Through experimental evolution, we identified two compensatory strategies that alleviate these defects: knockdown of ribosome-associated proteins (RsmA, RsmH, and RplI) that globally reduce decoding fidelity, and single-nucleotide deletions upstream of the internal stop codon that abolish autoregulation. We propose that the evolutionary dynamics of the internal stop codon hinge on its extreme tunability. Mutations in the PRF site tend to be deleterious, placing organisms on a sharp fitness peak where most mutations are harmful. In this view, loss of the internal stop codon may represent an evolutionary escape from this constraint.

## Results

### Characterization of *prfB* autoregulation in *P. fluorescens*

**Figure 2:**
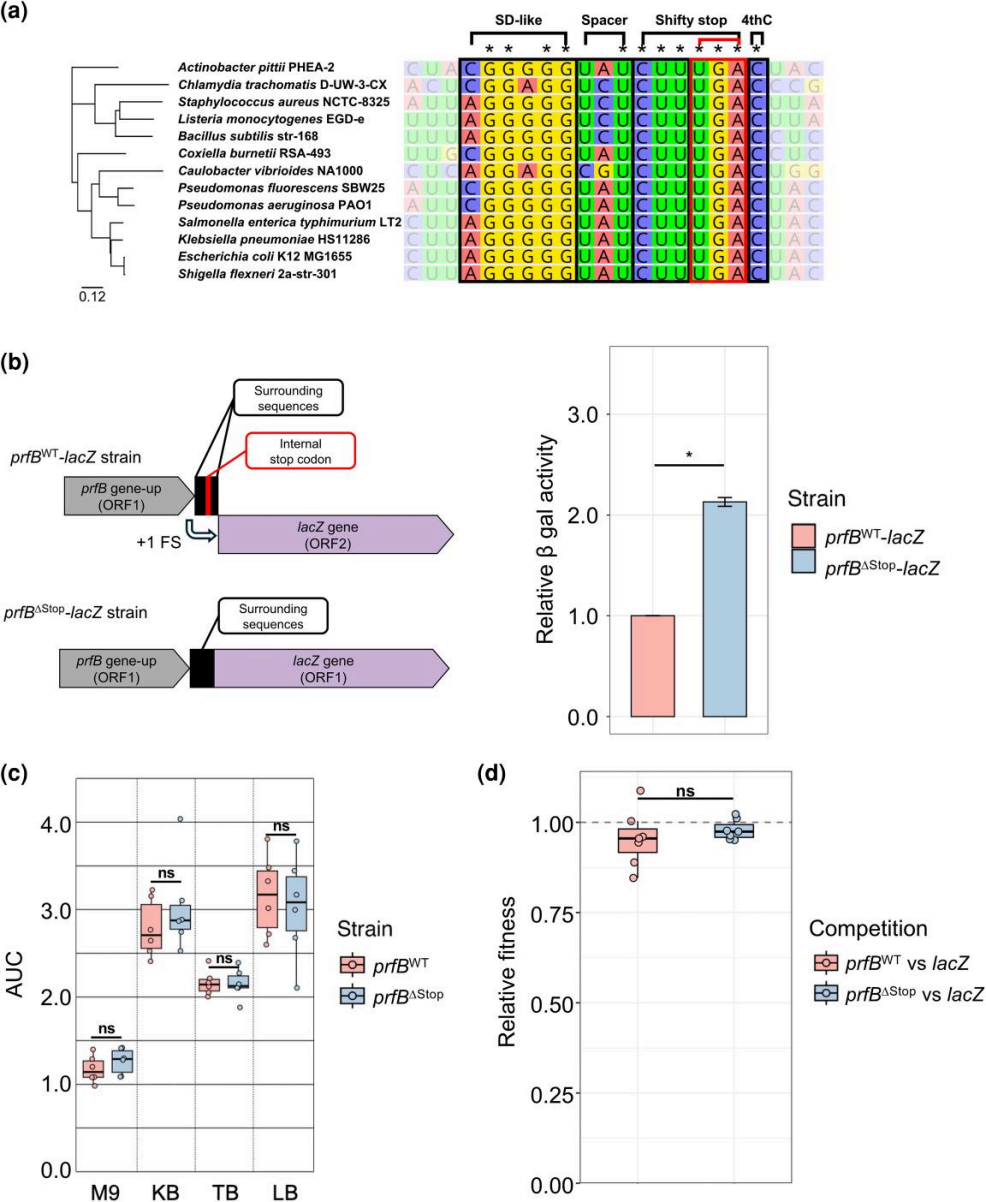
Characterization of PRF in *P. fluorescens prfB.* a) Nucleotide alignment of PRF sites from 13 bacterial species. Asterisks mark positions conserved across all sequences. The species tree (left) was inferred from 16S rRNA; the scale bar indicates branch length. Boxes highlight PRF-site features: black boxes denote flanking elements required for frameshifting (SD-like sequence, spacer codon, 4thC and shifty-stop sequence) and red boxes denote UGA stop codon. b) β-galactosidase activity of *prfB*^WT^-*lacZ* and *prfB*^ΔStop^-*lacZ* fusions. Left, schematics of the fusions: the *prfB* upstream fragment extending to the PRF site was placed upstream of *lacZ*, either retaining (WT) or removing (ΔStop) the first nucleotide of the internal UGA stop codon. Right, β-galactosidase activity was expressed relative to the wild type (*prfB*^WT^-*lacZ*). Data represent the average and standard error of five independent experiments. c) Area under the growth curve (AUC) for *P. fluorescens* strains carrying the wild-type *prfB* allele (*prfB*^WT^) or a mutant lacking the internal stop codon (*prfB*^ΔStop^). Cultures were grown in M9 minimal medium with 0.4% (w/v) Glucose (M9), King's broth (KB), Terrific broth (TB), or Lysogeny broth (LB). The value of each condition was represented as box plots. A horizontal line in the middle of the box indicates the median if the number of replicates is odd. If the number of replicates is even, the central two values are averaged and plotted as a horizontal line. The box covers the interquartile region (IQR) of each dataset, while the whiskers reach to the extreme individual value within 1.5 times the IQR from the median value. Each biological replicate (six in this case) is shown as an individual circle. This formatting on the box plot stays consistent across other box plots in the manuscript, while the number of replicates varies on each figure. d) Competition assays of *P. fluorescens* strains carrying the wild-type *prfB* allele (*prfB*^WT^) or the *prfB*^ΔStop^ allele against an isogenic reference strain bearing wild-type *prfB* and constitutive *lacZ* (labeled as *lacZ*) for colony discrimination on plates with X-gal. Assays were performed in King's Broth (KB). A relative fitness of 1 is indicated by the dashed line. Individual data points are shown as circles (n = 7 per group). “*” *P* ≤ 0.05; “ns” *P* > 0.05.

The *prfB* gene of *P. fluorescens* carries an internal stop after 24 codons, flanked by canonical PRF site elements: a SD-like sequence (CGG GGG), separated by a UAU spacer codon from a shifty CUU UGA stop, and a cytosine at the fourth position, immediately downstream of the stop codon (Fig. 2a). These elements likely determine frameshifting rate on PRF, through the mechanism illustrated in Fig. 1a.

To experimentally test the functionality of *P. fluorescens* PRF, we deleted the first nucleotide of the internal UGA stop codon (ΔStop), restoring the reading frame and bypassing the need for frameshifting to produce RF2. We quantified the impact of the deletion in RF2 production using two approaches. First, we generated translational *prfB-lacZ* fusions, in which *lacZ* was placed immediately after the fourth position, following the wild-type stop codon (*prfB*^WT^-*lacZ*), or the ΔStop allele (*prfB*^ΔStop^-*lacZ*). Both constructions were constitutively expressed and inserted at a neutral ectopic site in the *P. fluorescens* chromosome, between loci *pflu1179* and *pflu1180* (Zhang and Rainey 2007). β-Galactosidase activity measurements indicated that the lack of stop codon increased expression by more than twofold (Fig. 2b). Second, we raised an anti-RF2 antibody and directly measured the levels of RF2 in the wild type and in the *prfB*^ΔStop^ strain (Figure S1). The deletion of the internal stop codon resulted in a clear increase in RF2 levels, demonstrating that PRF-mediated autoregulation limits RF2 production in *P. fluorescens* SBW25.

**Figure 3:**
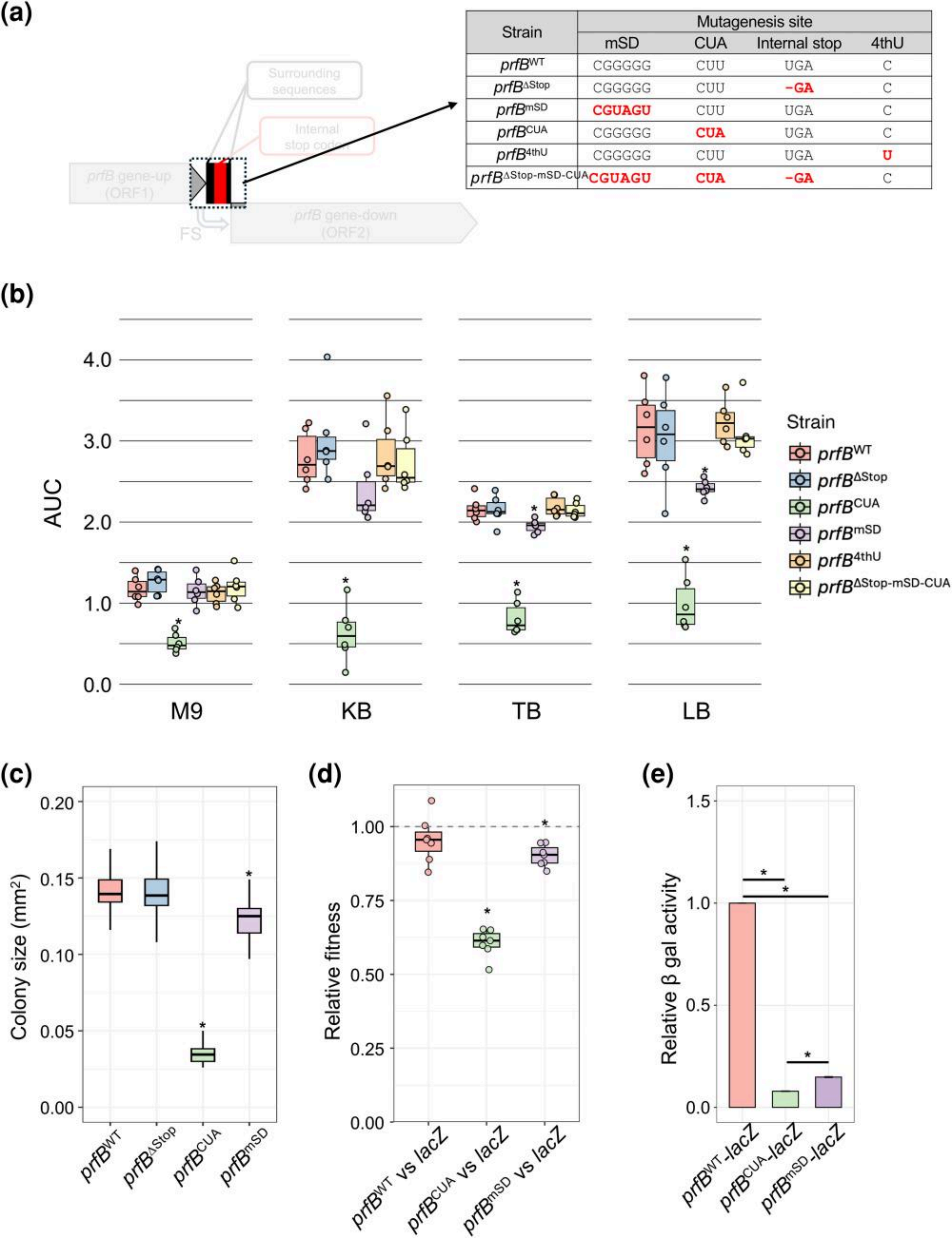
Characterization of mutations affecting conserved elements in the PRF site. a) Diagram of mutations introduced at the PRF site. The table lists each allele with its nucleotide changes; mutated elements are shown in red. b) Area under the growth curve (AUC) for *P. fluorescens* strains carrying the *prfB* alleles described in panel A. Cultures were grown in M9 minimal medium with 0.4% (w/v) Glucose (M9), King's broth (KB), Terrific broth (TB), or Lysogeny broth (LB). Box plots summarize six independent replicates (individual values shown as circles). See caption of Fig. 2c for box plot description. Values significantly lower than *prfB*^WT^ (*P* ≤ 0.05) are indicated with an asterisk. c) Colony size of strains carrying the indicated *prfB* alleles, after incubation on KB plates for 48 h at 28 °C. Colonies from three replicate plates were measured and averaged for each strain. Values significantly lower than *prfB*^WT^ (*P* ≤ 0.05) are indicated with an asterisk. d) Competition assays of *P. fluorescens* strains carrying the wild-type *prfB* (*prfB*^WT^), the *prfB*^CUA^ or the *prfB*^mSD^ allele against an isogenic reference strain bearing wild-type *prfB* and constitutive *lacZ* (labeled as *lacZ*) for colony discrimination on X-gal plates. Assays were performed in King's broth (KB). A relative fitness of 1 is indicated by the dashed line. Box plots summarize seven independent replicates (individual values shown as circles). Values significantly lower than *prfB*^WT^ vs *lacZ* competition (*P* ≤ 0.05) are indicated with an asterisk. e) β-galactosidase activity of *prfB*^WT^-*lacZ*, *prfB*^CUA^-*lacZ* and *prfB*^mSD^-*lacZ* fusions. Data were normalized to *prfB*^WT^-*lacZ*, and represent the average and standard error of five independent replicates. Statistically significant differences between value pairs are indicated with an asterisk.

We then assessed phenotypic consequences of removing *prfB* autoregulation. The *prfB*^ΔStop^ mutant grew similarly to wild type in different liquid media and formed colonies of equivalent sizes on plates (Figs 2c and 3c). We also competed *prfB*^WT^ and *prfB*^ΔStop^ strains against a *prfB*^WT^ strain constitutively expressing *lacZ* (for colony discrimination on plates with X-gal) in co-culture and found no competitive disadvantage associated with removing the stop codon (Fig. 2d). These results demonstrate that *P. fluorescens* carries a functional PRF system that mediates the autoregulation of RF2. Yet, elimination of the autoregulation has no detectable cost under the conditions tested.

### Fitness is impaired by mutations that reduce frameshifting rate

Next, we explored whether introduction of other mutations in the PRF site that might affect frameshifting rate caused noticeable phenotypes. We generated three additional variants (Fig. 3a): (i) messy Shine-Dalgarno (*prfB*^mSD^), in which the last two G nucleotides of each triplet were replaced by U (from CGG GGG to CGU GGU), (ii) a change in the leucine codon within the shifty-stop sequence from CUU to the rarer leucine codon CUA (*prfB*^CUA^), which recruits a different tRNA-Leu (anticodon UAG rather than GAG (Chan and Lowe 2016)) at the P-site and prevents wobble pairing with the +1 codon UUU, and (iii) a C-to-U substitution in the fourth position (*prfB*^4^th^U^). Importantly, none of these mutations alter the amino acid sequence of full-length RF2. Instead, they are predicted to affect termination efficiency at the internal stop codon and thereby modulate intracellular RF2 levels.

Two of the three modifications caused growth defects in liquid culture and produced smaller colonies on plates (Fig. 3b and c). The defects were strongest in the *prfB*^CUA^ mutant and more modest in the *prfB*^mSD^ mutant, whereas the *prfB*^4^th^U^ mutant showed no detectable defect. We confirmed these trends by competitions with the wild type in co-culture (Fig. 3d). We also observed that the strength of the growth defects was dependent on the medium (Fig. 3b). Hodges-Lehmann estimates comparing the wild type with each mutant (*prfB*^CUA^ and *prfB*^mSD^) in each medium supported this conclusion (Table S5). In minimal medium (M9), the estimated growth defect of the *prfB*^CUA^ mutant was milder (Hodges-Lehmann estimator, 0.65) than in rich medium (KB, 2.12; TB, 1.38; and LB, 2.15). In the case of the *prfB*^mSD^ mutant, no significant growth defect was observed in M9 (Table S5).

We reasoned that the reduced growth of the *prfB*^CUA^ strain, and to a lesser extent *prfB*^mSD^ strain, reflected decreased RF2 production, as both mutations were predicted to lower frameshifting rates. Consistent with this, attempts to combine the CUA and mSD mutations yielded no transformants, indicating that the combination is highly deleterious, but the double mutant was readily obtained and grew like wild type in a background lacking the internal stop codon (Fig. 3a and b). This result indicates that the codon changes introduced by the CUA and mSD mutations have no detectable effect on growth, and that the observed fitness defects are instead caused by reduced frameshifting, leading to decreased RF2 production. We confirmed reduced expression of RF2 using *lacZ* reporters and western blotting (Fig. 3e and Figure S1). In both mutants, RF2 protein levels dropped sharply and were barely detected by western blotting (Figure S1). In *lacZ* reporter mutants, *prfB*^mSD^-*lacZ* and *prfB*^CUA^-*lacZ,* β-galactosidase was also strongly reduced, with the *prfB*^CUA^-*lacZ* showing a larger decrease, in agreement with its stronger growth defect (Fig. 3e).

Overall, these results demonstrate that mutations that reduce frameshifting lower RF2 levels and reveal that the associated fitness costs are context-dependent, being more pronounced in rich medium than in minimal medium.

### Two types of mutations compensate the fitness defect caused by the *prfB*^CUA^ mutation

**Figure 4:**
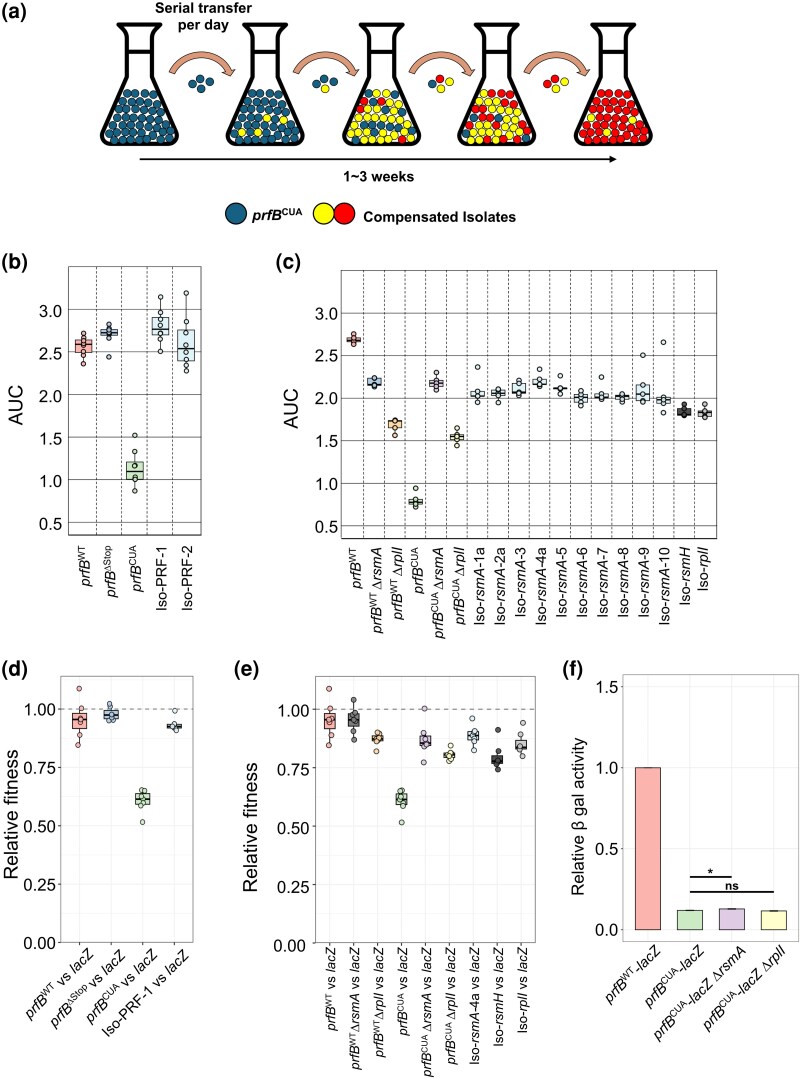
Isolation and characterization of mutations that compensate the *prfB*^CUA^ fitness defect. a) Diagram of the serial-transfer evolution experiment. Populations were transferred every 24 h at 1:100 or 1:1000 dilution. Blue circles, ancestral *prfB*^CUA^ strain; yellow and red circles, the compensated mutants. Further details are in the Supplementary Methods (“Evolution Experiment List”). b) Area under the growth curve (AUC) by 12 h for the following *P. fluorescens* strains: *prfB*^WT^*, prfB*^Δstop^*, prfB*^CUA^, and *prfB*^CUA^ with intragenic suppressor alleles isolated from the evolution experiment grown in KB medium. Data represents eight independent replicates. c) AUC for *prfB*^WT^ or *prfB*^CUA^, alone or combined with Δ*rsmA*, Δ*rplI*, or extragenic suppressor alleles, growing in KB medium. Data represents five independent replicates. d) Competition assays of *prfB*^WT^, *prfB*^ΔStop^, *prfB*^CUA^or *prfB*^CUA^ with a representative intragenic suppressor (Iso-PRF-1) against an isogenic *prfB*^WT^ reference strain bearing constitutive *lacZ* (labeled as *lacZ*) for colony discrimination on X-gal plates. Assays were performed in KB medium. A relative fitness of 1 is indicated by the dashed line. Data represents eight independent replicates. e) Competition assays of *prfB*^WT^ or *prfB*^CUA^, alone or with Δ*rsmA*, Δ*rplI*, or representative extragenic suppressor alleles, against the same reference. Data represents five independent replicates. f) β-galactosidase activity of *prfB*^WT^-*lacZ* and of *prfB*^CUA^-*lacZ* fusions in wild-type, Δ*rsmA* or Δ*rplI* backgrounds. Data were normalized to *prfB*^WT^-*lacZ*, and represent the average and standard error of five replicates. Statistically significant differences between value pairs are indicated with an asterisk.

We next investigated whether secondary mutations could suppress the fitness defect caused by the *prfB*^CUA^ mutation. To this end, we propagated the mutant strain in KB medium with daily transfers (Fig. 4a). Clones with increased fitness dominated the population after one to three weeks. We isolated a clone producing a colony similar in size to the wild-type strain from each evolving population after one week and sequenced their whole genomes to identify the compensatory mutations. To assess for parallelism in the outcomes, we repeated the experiment and sequenced candidate loci by Sanger sequencing (see Methods and Supplementary methods). In total, we characterized 34 isolates from independently evolved populations, which fell into two categories depending on whether the mutations were within the *prfB* PRF site (intragenic suppression, 4 isolates) or elsewhere in the chromosome (extragenic suppression, 30 isolates) (Table 1).

**Table 1:** Mutations increasing fitness of *prfB*^CUA^ isolated through experimental evolution.

Class	Isolate counts	Mutation details			
		Gene/Allele ID	DNA change^a^	Protein change	Predicted effects
Intragenic	2	*prfB*/Iso-PRF-1	(G)_5→4_ at position 62	Y23I, L24Y	Autoregulation elimination; two amino acid changes
Intragenic	2	*prfB*/Iso-PRF-2	ΔT at position 71	L24H	Autoregulation elimination; one amino acid change
Extragenic	2	*rsmA*/Iso-*rsmA-*1a	G133A	G45S	(i) Loss of 16S rRNA dimethylation (ii) Resistance to kasugamycin (iii) Decreased translational fidelity
Extragenic	1	*rsmA*/Iso-*rsmA*-1b	G134A	G45D	
Extragenic	1	*rsmA*/Iso-*rsmA*-2a	(G)_5→6_ at position 265	Frameshift	
Extragenic	1	*rsmA*/Iso-*rsmA*-2b	(G)_5→4_ at position 265	Frameshift	
Extragenic	1	*rsmA*/Iso-*rsmA*-3	Δ5 bp at position 445	Frameshift	
Extragenic	14	*rsmA*/Iso-*rsmA*-4a	(G)_6→7_ at position 452	Frameshift	
Extragenic	1	*rsmA*/Iso-*rsmA*-4b	(G)_6→5_ at position 452	Frameshift	
Extragenic	1	*rsmA*/Iso-*rsmA*-5	(G)_4→5_ at position 464	Frameshift	
Extragenic	1	*rsmA*/Iso-*rsmA*-6	(C)_3→4_ at position 539	Frameshift	
Extragenic	1	*rsmA*/Iso-*rsmA*-7	ins.7 bp at position 637	Frameshift	
Extragenic	1	*rsmA*/Iso-*rsmA*-8	ins.1277 bp at position 465	Disruption	
Extragenic	1	*rsmA*/Iso-*rsmA*-9	G121A	E41K	
Extragenic	2	*rsmA*/Iso-*rsmA*-10	G140A	G47D	
Extragenic	1	*rsmH*/Iso-*rsmH*	T309A	D103E	
Extragenic	1	*rplI*/Iso-*rplI*	(A)_6→7_ at position 186	Frameshift	

^a^Nucleotide position relative to the start codon of the mutated gene.

The intragenic suppressors harbored single-nucleotide deletions at the PRF site. Two isolates had a deletion of a G in the SD-like sequence, and two others a deletion of a T (U) immediately preceding the internal stop codon. Both types of mutations bypassed the internal stop codon and enabled RF2 production without the need for frameshifting. This compensation effectively abolished *prfB* autoregulation, resulting in elevated RF2 levels comparable to those of the *prfB*^ΔStop^ mutant (Figure S1), and restored growth and fitness to wild-type levels (Fig. 4b and d).

Most suppressors (30/34) were extragenic. The majority carried mutations in *rsmA* (also known as *ksgA*) (Table 1), a gene encoding a highly conserved 16S rRNA methyltransferase required for proper ribosome assembly (Pletnev et al. 2020). Loss of RsmA has been shown to confer resistance to the aminoglycoside antibiotic kasugamycin (Helser et al. 1972; Van Buul et al. 1984). These mutations included missense substitutions as well as multiple insertions and deletions that altered the reading frame (Table 1). Notably, 14 isolates carried a single-nucleotide expansion in a polyG tract in the middle of the gene, consistent with impaired RsmA function (Table 1). Accordingly, representative isolates were resistant to kasugamycin (Figure S2). In addition, one clone carried a single point mutation in *rsmH*, another 16S rRNA methyltransferase, resulting in a D103E amino acid substitution, and one clone carried a frameshift mutation in *rplI*, which encodes the ribosomal protein L9 (Table 1).

To evaluate the compensation efficiency of these mutations, we monitored growth of representative *rsmA*, *rsmH* and *rplI* isolates and performed competition assays against a wild-type ancestral constitutively expressing *lacZ* for colony discrimination on X-gal (Fig. 4c and e). As controls, we included *rsmA* and *rplI* deletion mutants (we were unable to obtain a *rsmH* deletion mutant). All mutations improved the growth and fitness of the *prfB*^CUA^ strain, with *rsmA* mutations providing the strongest rescue. However, both *rsmA* and *rplI* deletion decreased growth compared with the wild type, with *rplI* defect being more severe.

The inactivation of RsmA, RsmH or RplI has previously been reported to reduce translational fidelity and promote more frequent frameshifting on UGA stop codons (Van Buul et al. 1984; Herbst et al. 1994; Herr et al. 2001; Kimura and Suzuki 2010; Smith et al. 2019). We therefore hypothesized that these compensatory mutations restored growth of the *prfB*^CUA^ by increasing frameshifting rate at the PRF site of *prfB*, thereby increasing RF2 levels. To test this, we first measured RF2 levels by western blot in the *prfB*^CUA^ background with and without *rsmA* or *rplI* deletions. In all cases, RF2 levels remained low, barely above detection limit (Figure S1), suggesting that inactivation of RsmA or RplI did not substantially increase frameshifting rates to wild-type levels. We further explored this by measuring β-galactosidase activity using *prfB*^CUA^-*lacZ* fusions in wild type, RsmA^−^ or RplI^−^ backgrounds (Fig. 4d). Only a mild (but statistically significant) increase of β-galactosidase activity was observed in the *rsmA* mutant, and no increase in *rplI* mutant. Together, these results indicate that the rescue of the *prfB*^CUA^ strain by mutations in *rsmA* and *rplI* is not exclusively due to increased frameshifting at the PRF site of the *prfB* gene.

### Phylogenetic analysis reveals no significant correlation between genomic context and presence of PRF site in *prfB*

The observation that mutations abolishing *prfB* autoregulation are selected in mutants with reduced frameshifting rates (Fig. 4) is compatible with the idea that evolutionary loss of *prfB* autoregulation is favored when RF2 becomes limiting. In this context, previous proposals for the maintenance and loss of *prfB* autoregulation across bacterial species have emphasized correlations with genomic features. For example, high usage of the UGA stop codon has been proposed to favor autoregulation loss by increasing the demand for RF2 (Prince et al. 2025). Conversely, maintenance of autoregulation may be favored by features that increase the cost of RF2 overactivity. For instance, engineered RF2 alleles with enhanced ability to recognize UAG stop codons have also been shown to misrecognize UGG tryptophan codons in *S. enterica*, increasing premature termination at these codons (Abdalaal et al. 2020). Thus, high UGG codon usage could also contribute to the maintenance of *prfB* autoregulation in some cases.

**Figure 5:**
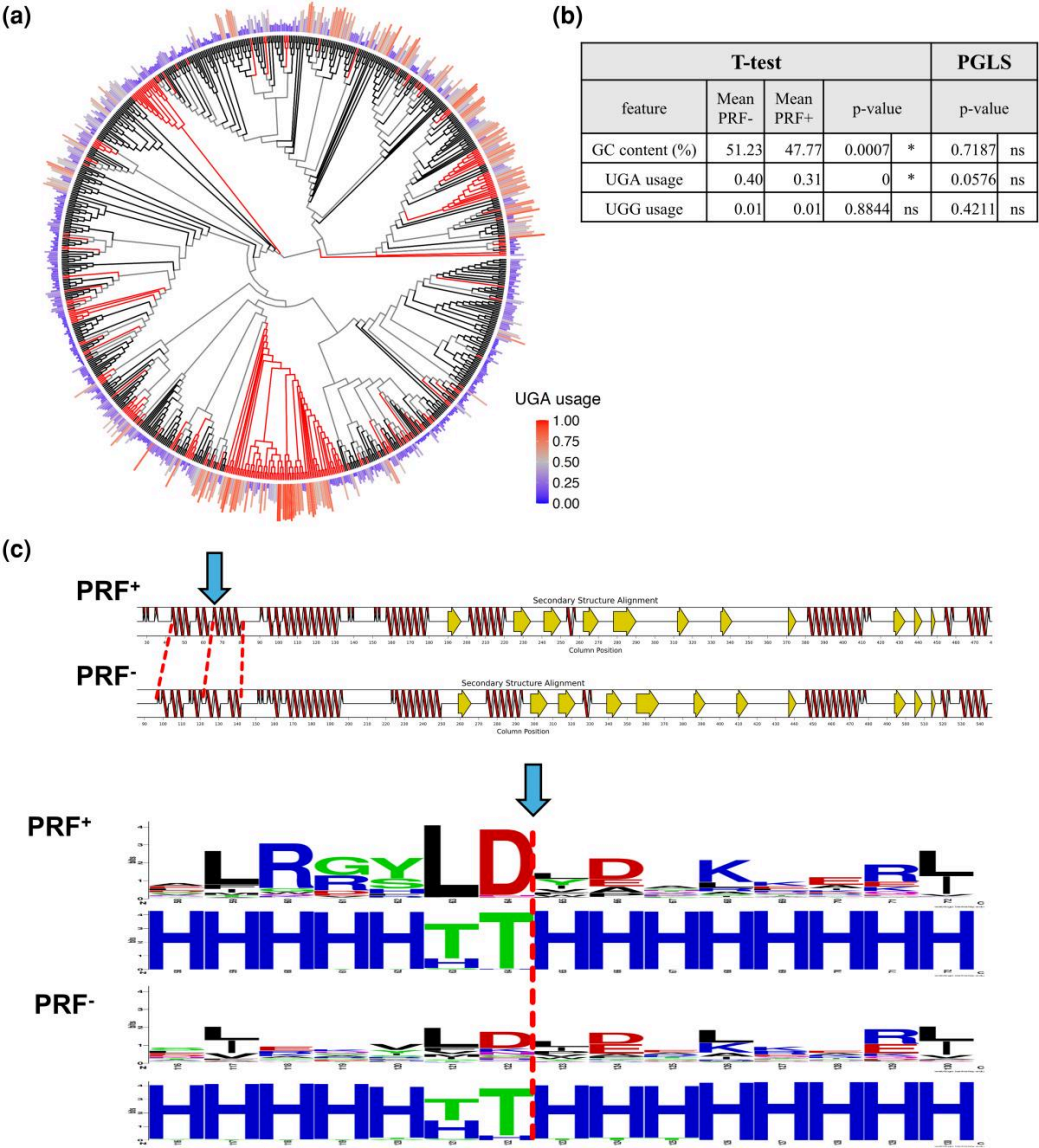
Computational analysis of PRF sites across bacterial species. a) Phylogenetic tree of 818 bacterial species. Leaves and branches for PRF^+^ species are black; PRF^−^ species are red. Species-level UGA stop codon usage is represented as a color-coded histogram, with a frequency of 0.5 shown in gray, higher frequencies in red, and lower frequencies in blue. The tree was built using UBCG2 (Kim et al. 2021). See Figure S4 for a high-resolution version of the tree including species names. b) Differences in GC content, UGA stop codon usage and UGG tryptophan codon usage between PRF^+^ and PRF^−^ species. For each feature, differences between PRF^+^ and PRF^−^ groups were tested with Welch's *t*-test and with Phylogenetic Generalized Least Squares (PGLS), a method that accounts for non-independence among species. Significance: * *P* ≤ 0.05; ns *P* > 0.05. *P*-values < 1 × 10^−4^ are displayed as 0. c) Predicted consensus secondary structure of the PRF region within RF2 in PRF^+^ species (*n* = 575) and of the equivalent region in PRF^−^ species (*n* = 243) (SIMSApiper v2) (Crauwels et al. 2024). Red lines, α-helices; yellow arrows, β-strands; black lines, loops or alignment gaps. The blue arrow marks the PRF site in PRF^+^ species. Red dashed lines connect the start and end of the first two α-helices in each group. Bottom, WebLogo plots derived from the corresponding amino-acid alignments (top for each group) and DSSP-based secondary-structure strings (bottom for each group); the blue arrow (PRF^+^) and red dashed line (PRF^−^) indicate the PRF site/orthologous region.

To explore these possibilities, we analyzed the genomes of 818 bacterial species from 35 phyla, including both species that retain (PRF^+^) and that have lost (PRF^−^) *prfB* autoregulation (Table S2 and S3) (sequences, including their corresponding PRF status, were kindly provided by Dr Fredrick (Naeem et al. 2023)). We reconstructed the phylogeny based on 81 core gene sequences (Kim et al. 2021), and mapped the PRF status of each species onto the tree (Fig. 5a). This analysis indicated that autoregulation loss occurred multiple times independently across bacterial phyla, consistent with previous reports (Naeem et al. 2023; Prince et al. 2025). However, autoregulation loss was not homogeneously distributed across the tree, but instead clustered within clades (Fig. 5a), suggesting that once lost, it was largely vertically inherited and that regain events were rare.

We then tested if PRF status correlates with genomic features that could influence the maintenance or loss of *prfB* autoregulation (Table S4). Specifically, we examined usage of the UGG tryptophan codon or the UGA stop codon. UGG usage was almost identical in PRF^+^ and PRF^−^ species, suggesting that it does not explain the retention or loss of autoregulation (Fig. 5b). However, PRF^+^ and PRF^−^ species differed in UGA usage: UGA accounted for 31% of stop codons in PRF^+^ species and ∼40% in PRF^−^ species, consistent with earlier observations (Fig. 5b) (Prince et al. 2025). While this pattern could suggest that increased UGA usage favors autoregulation loss, an alternative explanation is that it reflects phylogenetic clustering, as both PRF status and UGA usage tend to be conserved within clades (Fig. 5a). To account for phylogenetic relatedness, we applied phylogenetic generalized least squares (PGLS) analysis. This analysis reduced the strength of the association between UGA usage and PRF status and did not reach statistical significance at conventional thresholds (*P* = 0.0576) (Fig. 5b), indicating that the apparent correlation is largely explained by shared ancestry, while any remaining effect is at most weak. A close inspection of the tree supported this interpretation: while some autoregulation loss events were associated with higher UGA codon usage than neighboring PRF^+^ species (see, e.g., *Faecalibaculum rodentium* in the high-resolution tree with species names provided in Figure S3), this association was not universal, and we observed many cases of high-UGA species that retain autoregulation and, conversely, species with low UGA usage that have lost autoregulation.

## Discussion

Here, we have characterized PRF in the *prfB* gene of *P. fluorescens* and investigated the evolutionary dynamics of mutants with reduced frameshifting. Our data indicates that *prfB* autoregulation is dispensable under laboratory conditions, but that mutations that reduce frameshifting rate impose a context-dependent fitness cost, which is stronger in rich media than in minimal media. Using experimental evolution, we have found that bacteria can escape this constraint either by inactivating accessory ribosomal proteins or by a single-nucleotide deletion immediately upstream of the internal stop codon in *prfB*, which bypasses the need for PRF to produce full-length RF2.

These results suggest that increasing RF2 demand (or reduced RF2 availability) can act as a trigger for *prfB* autoregulation loss. We envision three non-mutually-exclusive scenarios where this could happen. First, higher RF2 demand may be imposed by the genomic context. One obvious possibility, in line with previous proposals (Wei et al. 2016; Ho and Hurst 2022; Prince et al. 2025), is that elevated usage of the UGA codon increases the cellular requirement for RF2 and thereby favors mutants in which autoregulation is eliminated. Under this view, lineages with greater reliance on UGA would experience more frequent or more severe bouts of RF2 limitation, making mutations that eliminate *prfB* autoregulation advantageous. A straightforward prediction is a positive association between autoregulation loss and UGA usage. Indeed, comparisons of PRF^+^ and PRF^−^ species show higher mean UGA usage in PRF^−^ lineages (Fig. 5b), consistent with earlier observations (Prince et al. 2025). However, when we accounted for shared ancestry using PGLS, this association was not significant (Fig. 5b), suggesting that elevated UGA usage alone does not impose a sufficiently strong or consistent RF2 demand to leave a robust phylogenetic signal in our dataset. Moreover, several PRF^−^ clades exhibit low UGA usage (Fig. 5a; Figure S3), indicating that additional evolutionary pressures must be at play. It remains possible, however, that the relevant signal is transcriptomic or locus-specific rather than genomic: for example, enrichment of UGA stops within a subset of highly expressed genes could create a substantial RF2 burden without shifting genome-wide averages, or the presence of an inefficient UGA in a particular gene could, when mistranslated, produce a toxic protein variant that perturbs physiology and intensifies selection for autoregulation loss. Such localized or transcriptomic pressures would be difficult to detect with global codon-usage summaries and may leave little phylogenetic trace, yet they could still provide the immediate genetic conditions that tip lineages toward the loss of *prfB* autoregulation. Conversely, the retention of *prfB* autoregulation in species with high-UGA codon usage might be explained if increased RF2 demand could be met by improving RF2 efficiency, rather by increasing RF2 levels through autoregulation loss.

A second scenario that could impose a higher requirement for RF2—and thereby favor autoregulation loss—is ecological. Exposure to particular antibiotics or toxic molecules can elevate termination demand. Aminoglycosides, for example, are known to increase stop codon readthrough (Diop et al. 2006; Malik et al. 2010), a defect that could be ameliorated by higher levels of translation termination factors. In this sense, it has been recently reported that a *Flavobacterium johnsoniae* mutant lacking *prfB* autoregulation exhibits a modest growth advantage when exposed to sublethal concentrations of streptomycin (Naeem et al. 2023), suggesting that increased RF2 levels can contribute to aminoglycoside resistance in at least some contexts. Consistent with this, we observed that the lack of *prfB* autoregulation causes a modest increase in resistance to the aminoglycosides, streptomycin and gentamicin in *P. fluorescens* SBW25 (Figure S4). In contrast, no significant change was observed for chloramphenicol, which inhibits peptide elongation thus stalling translation elongation, indicating that this phenotype is aminoglycoside-specific (Figure S4). More broadly, ecological regimes that entail sustained elevations in global gene expression, such as frequent nutrient upshifts or oscillatory feast-famine cycles, could also select for higher RF2 levels by increasing overall translation flux, thereby creating periods of RF2 limitation and, in turn, favoring the loss of autoregulation.

The third scenario that may favor autoregulation loss is analogous to our evolution experiment: the gradual accumulation of mutations in the *prfB* PRF element that lower frameshifting efficiency, whose fitness costs can be suppressed by eliminating autoregulation altogether. Such mutations could be nearly neutral when the translational burden is low, for example during slow growth. Indeed, our results show that the growth defects caused by the *prfB*^CUA^ and *prfB*^mSD^ alleles are smaller in minimal than in rich medium, with *prfB*^mSD^ showing no defect in minimal medium (Fig. 3b), suggesting that the fitness cost associated with lower RF2 levels is context dependent. However, if conditions change and translation rate increases, low-frameshifting mutants may not be able to cope with the increased translation termination demand, leading to the selection of suppressors that restore fitness. As shown in our experiments, some of these suppressors are single-nucleotide deletions upstream of the internal stop codon in *prfB*, which bypass the internal stop codon and eliminate autoregulation.

Any of the three scenarios outlined above could increase RF2 demand, thereby favoring autoregulation loss. However, PRF itself may be under direct positive selection in some species or ecological contexts, as it enables cells to tune RF2 abundance to match translational demands within a certain range. In this sense, overproduction of RF2 has been shown to be detrimental in some species under certain laboratory conditions (Jørgensen et al. 1993; Rengby and Arnér 2007; Abdalaal et al. 2020; Lalanne et al. 2021; Prince et al. 2025), and the same is likely true in natural environments. Thus, autoregulation loss via the compensatory routes we describe should be selectively favored only when increased RF2 availability is strongly beneficial (e.g., under RF2-limiting conditions) and the fitness costs of RF2 overproduction and loss of autoregulation are comparatively small.

In PRF^‒^ species, the nucleotide and amino acid sequences surrounding the PRF site (from the SD-like sequence to the internal stop codon) are degraded (Prince, Lin and Feaga 2025) (Fig. 5c). This sequence divergence under drift likely hinders the regain of autoregulation, because it is expected to disrupt elements required to sustain high frameshifting rates. Thus, regain events, if any, would most likely occur shortly after loss, before the surrounding PRF region decays, or via horizontal gene transfer from a PRF^+^ donor. Despite the decay of the amino acid sequence, the predicted secondary structure—consisting of two initial α-helices and the intervening loop—is retained, suggesting that this structural element is under selection independently of PRF status. The position of the PRF site in a turn between two α-helices may minimize its impact on RF2 structure and co-translational folding, allowing the autoregulatory sequence to evolve with limited consequences for RF2 function (Worth et al. 2009).

In addition to eliminating *prfB* autoregulation, our evolution experiment repeatedly produced mutations that inactivated or reduced the function of the accessory ribosomal proteins RsmA, RsmH, and RplI (Table 1). Although loss of these proteins is not an optimal solution in our experimental conditions, as evidenced by the reduced fitness of the Δ*rsmA* and Δ*rplI* mutants relative to wild type (Fig. 4c), such mutations arose in many independent lineages, suggesting they are highly accessible. Mutations in *rsmA* were especially frequent. In particular, indels in poly-G tracts within the coding sequence caused frameshifts and complete loss of RsmA function (Table 1). This pattern raises the possibility that *rsmA* is a mutational hotspot. Such a bias might have an evolutionary explanation: RsmA deficiency confers resistance to the antibiotic kasugamycin (Helser et al. 1972; Van Buul et al. 1984) (Figure S2) and can ameliorate genetic perturbations that impair translation initiation (Das et al. 2008). Since poly-G tracts are prone to slippage (Levinson and Gutman 1987; Burch et al. 1997), the ability to expand or contract these tracts could create locally elevated, reversible mutability, allowing transient inactivation of *rsmA* during periods of translational stress and restoration of function once the perturbation subsides.

The mechanisms underlying suppression of the *prfB*^CUA^ growth defect by mutations in *rsmA*, *rsmH* and *rplI* remain unclear. Inactivation of RsmA or RplI produced only minor or negligible increases in *prfB* frameshifting (Fig. 4f; Figure S1), indicating that suppression is unlikely to result from restoration of sufficient frameshifting rate and consequent elevation of RF2. An alternative, non-exclusive explanation is that loss of these accessory factors increases the probability of bypassing UGA stops (i.e. enhanced readthrough or miscoding at termination). This explanation may be the case particularly relevant for *rplI* mutations, which have previously been reported to increase ribosome slippage and thereby facilitate frameshifting (Herbst et al. 1994; Herr et al. 2001; Smith et al. 2019), and for *rsmA* mutations, which have been reported to increase leakiness to nonsense and frameshift mutations (Van Buul et al. 1984). Under low-RF2 conditions, ribosomes are expected to dwell abnormally long at UGA codons, creating queues that sequester ribosomes and limit recycling for new rounds of initiation. If inactivation of RsmA or RplI raises the rate of UGA bypass (or otherwise reduces dwell time at problematic stops) this would lessen ribosome queuing, increase the pool of recyclable ribosomes, and partially restore growth despite persistent RF2 scarcity. However, these mutations are likely to be beneficial only in the *prfB*^CUA^ background, where they alleviate the consequences of RF2 limitation, whereas in a wild-type background they may impose costs by perturbing the translational landscape. This tradeoff may explain why they only partially restore fitness. More generally, by relieving ribosome queuing, both *rsmA* and *rpI* mutations may also mitigate secondary consequences of RF2 depletion on global mRNA stability. Specifically, because translating ribosomes protect bacterial mRNAs from degradation (Régnier and Arraiano 2000; Müller et al. 2025; Zhang et al. 2025), ribosomes trapped at UGA codons on a subset of transcripts would otherwise deplete ribosome density elsewhere, rendering many mRNAs more prone to decay.

This model is more complex for *rsmA* mutants, however, because the 16S rRNA methylations at positions A1518 and A1519 mediated by RsmA have also been implicated in ribosome recycling (Seshadri et al. 2009). Thus, RsmA inactivation could, on the one hand, increase ribosome availability by promoting UGA stop-codon bypass, while, on the other hand, reducing the overall efficiency of ribosome recycling because of the absence of specific 16S rRNA methylations. Whether the net outcome is an increase or decrease in available ribosomes would depend on the balance between these opposing effects.

A second, complementary possibility is a general effect on translation capacity: RsmA loss perturbs 30S maturation and initiation fidelity (Sharma and Anand 2019), and RplI influences elongation dynamics and quality control (Herbst et al. 1994). The inactivation of either protein could reduce effective initiation throughput or alter elongation kinetics enough to lower the overall demand on termination, thereby rebalancing translational demand and RF2 availability. Together, these effects would relieve the bottleneck created by RF2 limitation without directly restoring frameshifting. Further work is necessary to explore these mechanisms.

Although the models described above could apply to *rsmA* and *rplI* mutations, the situation is more puzzling for *rsmH*. Loss-of-function mutations in RsmH have been reported to reduce UGA stop-codon readthrough and +1 frameshifting (Kimura and Suzuki 2010), which would, in principle, be expected to exacerbate rather than alleviate the defects caused by the *prfB*^CUA^ mutation. The *rsmH* allele that evolved in our suppression experiments encodes an aspartate-to-glutamate substitution at position 103, which may modulate RsmH activity rather than abolish it. Nevertheless, whether this altered RsmH activity can account for suppression, or whether *rsmH* mutations have other uncharacterized phenotypic effects that contribute to suppression of the *prfB*^CUA^ phenotype, remains to be determined.

Overall, our results clarify the mechanisms of RF2 autoregulation in *P. fluorescens* and show how changes in RF2 demand, coupled with compensatory mutations in *prfB*, can drive the loss of autoregulation. Altogether, this work helps explain the maintenance and repeated loss of *prfB* autoregulation across bacterial lineages.

## Materials and methods

### Strains and growth conditions

All the strains used in this study are derivatives of *P. fluorescens* SBW25 (NCBI Reference Sequence: NC_012660.1) (Silby et al. 2009). Strain and oligonucleotide lists are provided as Table S1. All mutants were constructed using scar-free two-step allelic exchange protocol using pUI*sacB*, as previously described (Farr et al. 2025). King's medium B (KB) (King et al. 1954), Lysogeny broth (LB), Terrific broth (TB) or M9 minimal media with 0.4% (w/v) Glucose were used for culturing bacteria, depending on the experiment. Strains were routinely grown on KB agar plates at 28 °C for 48 h. Liquid cultures were grown overnight at 28 °C in an orbital shaking incubator at 220 rpm.

### β-Galactosidase assays

β-Galactosidase assays were performed as previously described (Raval et al. 2023), with modifications for strains with low *lacZ* expression. To increase sensitivity, we extended the reaction time up to 24 h and concentrated overnight cultures fivefold. Z buffer (Na_2_HPO_4_ 60 mM, NaH_2_PO_4_ 40 mM, KCl 10 mM, MgSO_4_ 1 mM, 5% β-mercaptoethanol) was freshly prepared before each assay. Chloramphenicol (100 µl, 3 mg/ml) was added to 500 µl of concentrated overnight culture to block translation. After 10 min on ice, 350 µl of Z buffer was added and incubation on ice was continued for 1 h. The reaction was initiated by adding 200 µl of *o*-nitrophenyl-β-D-galactopyranoside (ONPG) solution (12 mg/ml) and incubating at 30 °C. At each time point, 50 µl of the reaction mixture was withdrawn and the reaction was stopped by adding 25 µl of 1 M Na_2_CO_3_. After centrifugation (12,000 × *g*, 1 min), 50 µl of the supernatant was transferred to a clear 96-well plate and OD_420_, OD_550_ and OD_600_ were measured. Because of the long incubation times, β-galactosidase activity was compared directly using OD_420_ values after confirming the absence of cell debris in the supernatant by OD_550_ and OD_600_ measurements.

### Growth curves and competition assays

For growth curves, overnight precultures were prepared in 96-well plates by inoculating individual colonies into 200 µl of growth medium per well. Then, 2 µl of each preculture were inoculated into 198 µl of fresh growth medium in a 96-well plate. OD_600_ was recorded every 10 min, with orbital shaking (282 rpm, 3 mm amplitude) for 5 s before each measurement. To calculate area under the growth curve (AUC), we used R package, “Growthcurver” with version 0.3.1, using parameter “auc_e” (Sprouffske and Wagner 2016). The individual growth curves used to calculate AUC are provided in Figure S5.

Competition assays were performed as described previously (Khomarbaghi et al. 2024), in four experimental blocks with three replicate competitions per strain pair. Precultures were prepared as above. After overnight growth, the two competitors for each pair were mixed at an approximate 1:1 ratio, and 4 µl of this mixture were used to inoculate 4 ml KB in a 13-ml tube, which was then incubated for 24 h (28 °C, 220 rpm). Samples were taken at the start (T0) and end (T24) of the competition, serially diluted, and plated on LB agar containing 60 µg ml^−1^ X-gal (48 h, 28 °C). Colonies of each competitor were counted at T0 and T24, with strains distinguished by colony color as one of the competitors was the SBW25-*lacZ* strain (Zhang and Rainey 2007), which expresses *lacZ* constitutively. Changes in competitor ratios were used to calculate relative fitness as described previously (Khomarbaghi et al. 2024).

### Colony size measurement

To measure the colony size of wild-type and mutant strains, dilutions from overnight cultures were spread onto KB agar plates to obtain between 10 and 50 colonies per plate. The plates were incubated for 48 h at 28 °C. After incubation, images of the plates were captured, and colony sizes were quantified using ImageJ (version 1.54p). For quantification, images were converted to 8-bit grayscale and segmented using a fixed pixel intensity threshold of 75 to 255. We filtered out the segmentations below 0.015 mm^2^ and applied circularity filter of 0.80 to 1.00 to exclude non-colony artifacts.

### Serial transfer evolution experiment

Individual colonies of the *prfB*^CUA^ strain were used to inoculate 4 ml KB medium in 13-ml tubes to generate precultures. For each transfer, 40 µl or 4 µl of preculture were inoculated into 4 ml fresh KB medium in a new 13-ml tube, and each lineage was subsequently transferred with the same volume into fresh medium every 24 h. Colony Forming Units (CFU) assays were performed on day 1 and then weekly to monitor contamination and the appearance of compensatory mutations (assessed by colony size). Cryo-stocks of the evolving populations were prepared every week and stored at −80 °C. From the weekly CFU plates, a single colony per lineage was picked after 48 h incubation at 28 °C. When colonies of different sizes were present, we selected the largest colony; otherwise, a colony of median size was chosen. As a control for contamination, a fresh tube containing only KB medium was carried through the daily transfer regime.

### Sequencing

Evolved isolates showing significant growth improvement were first Sanger-sequenced across the *prfB* coding region. Isolates without mutations in *prfB* were then subjected to whole genome sequencing (detailed in the next paragraph). After *rsmA* was identified as a recurrent mutational target in the evolution experiment, both *prfB* and *rsmA* were Sanger-sequenced in all selected evolved isolates. Whole-genome sequencing was additionally performed for isolates that did not carry mutations in both gene. Primers used for Sanger sequencing are listed in Table S1. Geneious Prime (v 2025.1.3) was used to identify mutation sites.

Six isolates obtained from the evolution experiment were whole-genome sequenced (*rsmA*-1a-1, *rsmA*-2b, *rsmA*-4a-1, *rsmA*-5, *rsmH*-1, *rplI*-1). Library preparation and sequencing were performed by Novogene Europe (Cambridge, UK) using Illumina technology on a NovaSeq 6000 platform. For four isolates (*rsmA*-1a-1, *rsmA*-2b, *rsmA*-4a-1, *rsmA*-5), 150-bp paired-end reads were generated. For the remaining two isolates (*rsmH*-1, *rplI*-1), 250-bp paired-end reads were generated with an Illumina MiSeq instrument at the Max Planck Institute for Evolutionary Biology (Plön, Germany) using standard procedures (Khomarbaghi et al. 2024). A minimum of 0.575 million raw reads per genotype were obtained and aligned to the SBW25 reference genome (NC_012660.1) (Silby et al. 2009) using breseq (Deatherage and Barrick 2014) and Geneious Prime (v 2024.0.2).

### Western blotting

For each strain, a single colony from a 48-h plate was used to inoculate culture in 4 ml of KB medium and grown overnight at 28 °C in an orbital shaking incubator at 220 rpm. One ml of overnight culture was adjusted to OD_600_ = 2.0, centrifuged (12,000 × *g*, 1 min), and the pellet resuspended in 200 µl sample preparation buffer (100 µl 2× Laemmli buffer, Bio-Rad #1610737; 95 µl distilled water; 5 µl β-mercaptoethanol). Samples were incubated at 95 °C for 5 min and centrifuged again (12,000 × *g*, 10 min).

Stacking (4% polyacrylamide) and running (10% polyacrylamide) gels were cast following the Handcasting Polyacrylamide Gels protocol (Bio-Rad Bulletin 6201) and mounted in a Mini-PROTEAN Tetra Vertical Electrophoresis Cell (Bio-Rad #1658004). Fifteen µl of each sample or protein ladder (Bio-Rad #1610376) were loaded per lane. Electrophoresis was carried out at 30 V for 30 min followed by 70 V for 150 min. Proteins were then transferred to a PVDF membrane (Bio-Rad #1704156) using the Trans-Blot Turbo Transfer System (Bio-Rad #1704150).

Membranes were blocked for 1 h at room temperature in 5% skim milk in Tris-buffered saline with 0.1% Tween 20 (TBST), then incubated overnight at 4 °C with primary antibody diluted to a final concentration of 0.5 µg/ml in 5% skim milk in TBST. The primary antibody was a rabbit polyclonal antibody raised against a *P. fluorescens* SBW25 RF2 antigenic peptide (CVRKSPFDSGNRRHT) by GenScript. After five 10-min washes in TBST, membranes were incubated for 1 h at room temperature with secondary antibody (goat anti-rabbit IgG–HRP, Bio-Rad #1721019) diluted 1:3000 in 5% skim milk in TBST, followed by five additional 10-min washes in TBST. All incubation and rinsing steps were performed with gentle agitation at ∼30 rpm. Chemiluminescent substrate (Abcam #ab133406) was applied for 2 min before imaging, and signals were captured using a Bio-Rad ChemiDoc Imaging System.

### Phylogenetic analyses

RF2 amino acid sequence and its PRF status was kindly provided by Dr Fredrick's group at Ohio State University (Naeem et al. 2023). A database of 4971 microbial genomes was downloaded from NCBI genome dataset filtered with (i) reference genomes, (ii) annotated genomes, and (iii) assembly level: Complete at 04.2023. Detailed species list is provided in Table S2. The genome database was dereplicated using dRep v.3.5.0 to reduce redundancy (over-representation of closely related genomes) and to filter for high-quality genome assemblies (Olm et al. 2017). Filtering was performed with the following criteria: Completeness ≥ 90% and contamination ≤ 1% (ran by CheckM taxonomy workflow); primary clustering threshold of 70% ANI; secondary clustering threshold of 50% ANI using the fastANI algorithm. The list of genomes retained at each filtering step, together with the full filtering workflow, is provided in Table S3.

Phylogeny was reconstructed based on 81 core genes, using the UBCG2 pipeline (Kim et al. 2021). Weblogo image showing amino acids and secondary structures at the PRF site was constructed based on an alignment of RF2 amino acid sequences generated with SIMSApiper v2, using Weblogo webtool (https://weblogo.berkeley.edu/logo.cgi).

PGLS analysis was performed to test whether PRF status is associated with genomic features such as codon usage and GC content. PRF status was used as the predictor variable, coded as 0 for PRF− and 1 for PRF+. The following response variables were tested: (i) GC content (percentage, 0 to 100), (ii) UGA stop codon usage (relative frequency among the three stop codons, 0 to 1), and (iii) UGG codon usage (frequency relative to total sense codons, 0 to 1). Feature values for each species are provided in Table S4. PGLS was performed using the phylolm package under a Brownian motion evolutionary model. For non-phylogenetic comparisons, Welch's two-sample *t*-tests (unequal variances) were used. Statistical significance was defined as *P* < 0.05. Version information for the software and packages used for phylogenetic analyses were as follows: R version: 4.4.1 (2024-06-14); R package(“phylolm”): 2.6.5; R package(“ape”): 5.8.1; R package (“ggplot2”): 3.5.1.

### Statistical analyses

All statistical analyses were performed in R version 4.4.1 (2024-06-14), using the “stats” package. Unless otherwise stated, differences between groups were assessed with Welch's *t*-test. Normal *P*-values were reported for single pairwise comparisons while the Benjamini-Hochberg adjustment was applied for multiple comparisons. For the competition assays, we used a one-way ANOVA to test for differences among competition groups. Residuals violated normality (Shapiro–Wilk *W* = 0.952, *P* = 0.00337) while variances were homogeneous (Levene's test *F* = 0.82, *P* = 0.62). Pairwise comparisons were then performed using Dunn's test with Bonferroni correction to obtain adjusted *P*-values.

## Supplementary Material

msag125_Supplementary_Data

## Data Availability

All raw experimental data including sequencing data can be found in Zenodo (doi:10.5281/zenodo.18065983).
