# Supplementary material for: Compensatory evolution facilitates loss of prfB autoregulation in Pseudomonas fluorescens SBW25
Source: Mol Biol Evol. 2026 May 26;43(6):msag125. doi: 10.1093/molbev/msag125 (PMC13245932; doi:10.1093/molbev/msag125)
Supplement: msag125_Supplementary_Data [file msag125_supplementary_data.zip › Lim_Supplementary-Information.pdf]

Supplementary information for

Compensatory evolution facilitates loss of *prfB* autoregulation in *Pseudomonas fluorescens* SBW25

Sungbin Lim, Frederic Bertels, Javier Lopez-Garrido* and Jenna Gallie*†
Max Planck Institute for Evolutionary Biology, 24306 Plön, Germany

*Correspondence: lopezgarrido@evolbio.mpg.de and jenna@cellora.bio
†Current address: Cellora, Pōkeno 2402, New Zealand

Content:

- Supplementary Figures S1 to S5
- Supplementary Methods

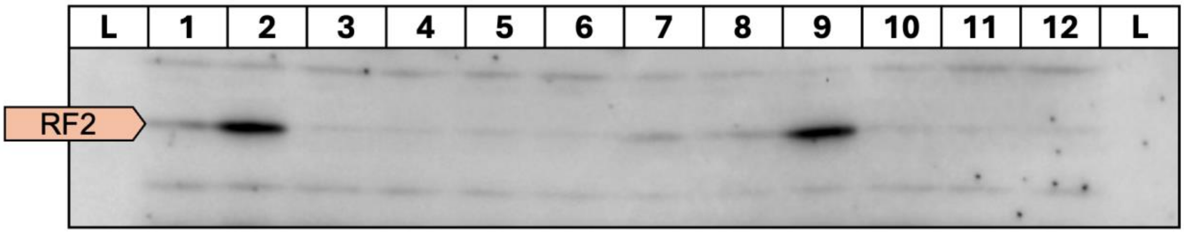

Supplementary Figure S1. Western blot analysis of RF2 in wild-type *Pseudomonas fluorescens* SBW25 and in different mutant strains. The RF2 band (expected size: 41 kDa) is indicated by an arrow box. Nonspecific bands are also observed above and below the RF2 band. The image was cropped to display target region. The image was taken with a 300-second exposure. Full images including alternative exposure times are provided in the Supplementary Data. Lane annotations are as follows:

L – Protein ladder
1 – *prfB*^{WT}
2 – *prfB*$^{\Delta Stop}$
3 – *prfB*^{CUA}
4 – *prfB*^{mSD}
5 – *prfB*^{CUA} Δ*rsmA*
6 – *prfB*^{CUA} Δ*rplI*
7 – Δ*rsmA*
8 – Δ*rplI*
9 – *prfB*^{CUA} Iso-PRF-1
10 –*prfB*^{CUA} Iso-*rsmA*-4a
11 –*prfB*^{CUA} Iso-*rsmH*
12 –*prfB*^{CUA} Iso-*rplI*
L – Protein ladder (duplicate)

Alt Text: Supplementary Figure S1 shows a Western blot comparing RF2 protein levels in wild-type *Pseudomonas fluorescens* SBW25 and several mutant strains affecting *prfB* autoregulation. The expected RF2 band is marked at about 41 kDa, while additional non-specific bands are visible above and below. Each lane contains a different strain, including the wild type, strains with mutations in the *prfB* frameshifting site, strains carrying deletions in *rsmA* or *rplI*, and evolved suppressor strains. The figure is used to compare how these mutations affect RF2 abundance.

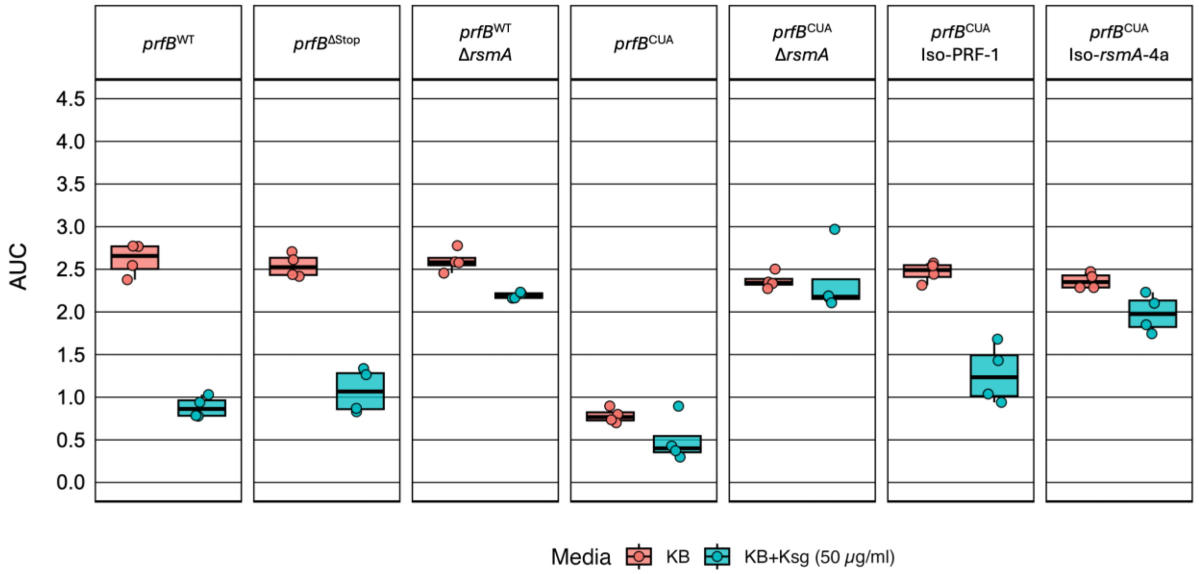

Supplementary Figure S2. Impact of mutations in *prfB* and *rsmA* on kasugamycin resistance. Area under the growth curve (AUC) during 12 hours of growth with the indicated *P. fluorescens* strains (top of each panel) in KB medium in the absence (red) or in the presence (green) of 50 µg/ml kasugamycin (Ksg). Representative evolved isolates were included in the analysis. Data represents four independent replicates. See the legend of Figure 2C for box plot description.

Alt Text: Supplementary Figure S2 compares growth of different *Pseudomonas fluorescens* strains in the presence and absence of the antibiotic kasugamycin. Each panel shows one strain, including mutants in *prfB* and *rsmA* as well as representative evolved isolates. Growth is reduced by kasugamycin in some strains but less affected in those lacking a functional version of RsmA.

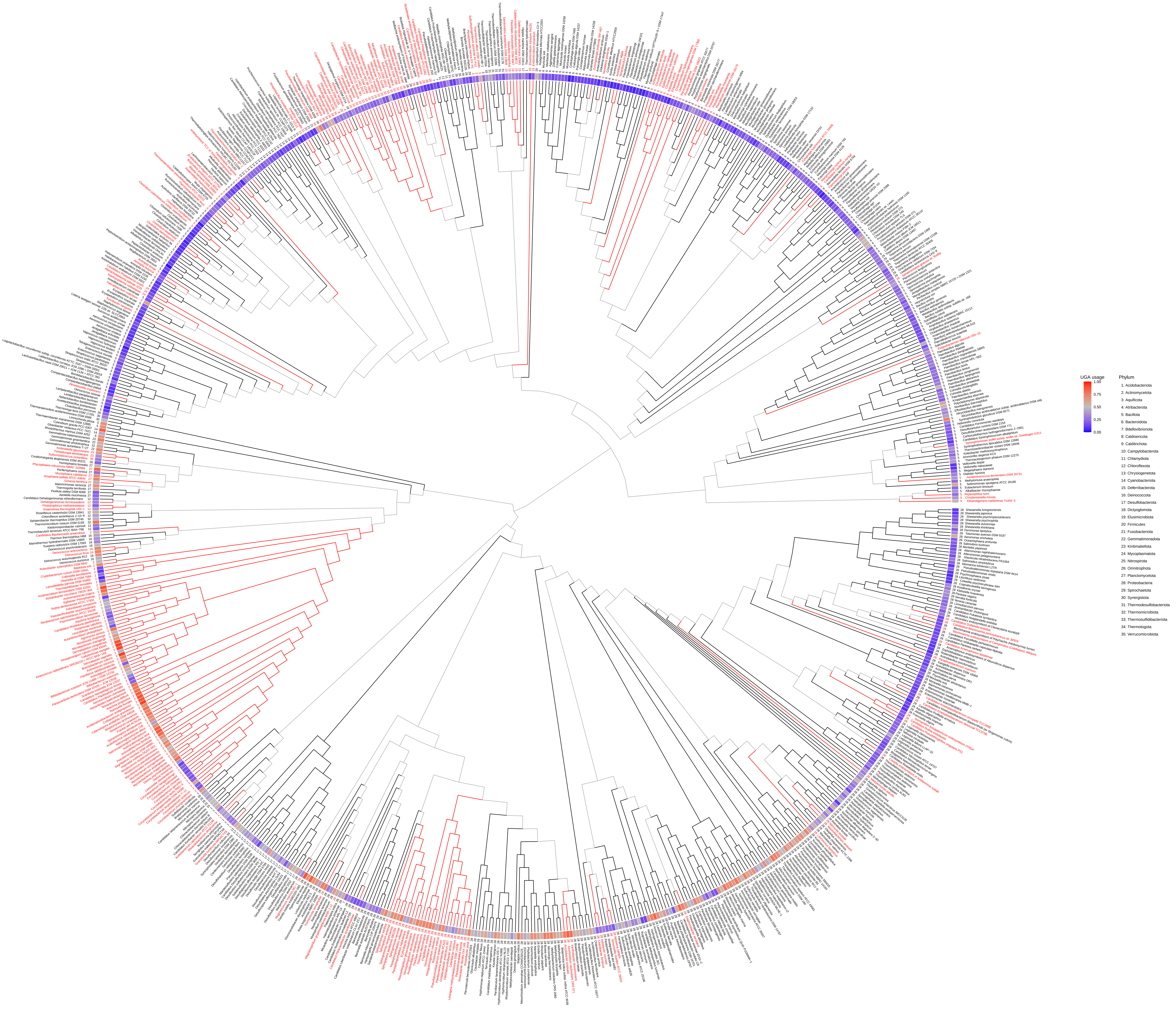
UGA usage
1.00
0.75
0.50
0.25
0.00
Phylum
1: Acidobacteriota
2: Actinomycetota
3: Aquificota
4: Atribacterota
5: Bacillota
6: Bacteroidota
7: Bdellovibrionota
8: Caldisericota
9: Calditrichota
10: Campylobacterota
11: Chlamydiota
12: Chloroflexota
13: Chrysiogenetota
14: Cyanobacteriota
15: Deferribacterota
16: Deinococcota
17: Desulfobacterota
18: Dictyoglomota
19: Elusimicrobiota
20: Firmicutes
21: Fusobacteriota
22: Gemmatimonadota
23: Kiritimatiellota
24: Mycoplasmatota
25: Nitrospirota
26: Omnitrophota
27: Planctomycetota
28: Proteobacteria
29: Spirochaetota
30: Synergistota
31: Thermodesulfobacteriota
32: Thermomicrobiota
33: Thermosulfidibacteriota
34: Thermotogota
35: Verrucomicrobiota

Supplementary Figure S3. High-resolution phylogenetic tree based on core gene sequence with species name indicated (A) Phylogenetic tree based on core gene sequence (818 species) with high-resolution including species name on the tip of each node. Leaves and branches for PRF^{+} species are black; PRF^{-} species are red. Species-level UGA stop codon usage is represented as a color-coded heatmap, with a frequency of 0.5 shown in gray, higher frequencies in red, and lower frequencies in blue. The tree was built with UBCG2 (Kim *et al.* 2021). Numbers at the tip of each branch indicates the phyla to which each species is assigned, according to NCBI.

Alt Text: Supplementary Figure S3 shows a high-resolution phylogenetic tree of 818 bacterial species, with the name of each species labeled. Species that retain *prfB* programmed frameshifting are shown in black, and species that have lost it are shown in red. A color scale next to the tree indicates UGA stop-codon usage for each species, allowing comparison of frameshifting status and codon usage across the bacterial phylogeny.

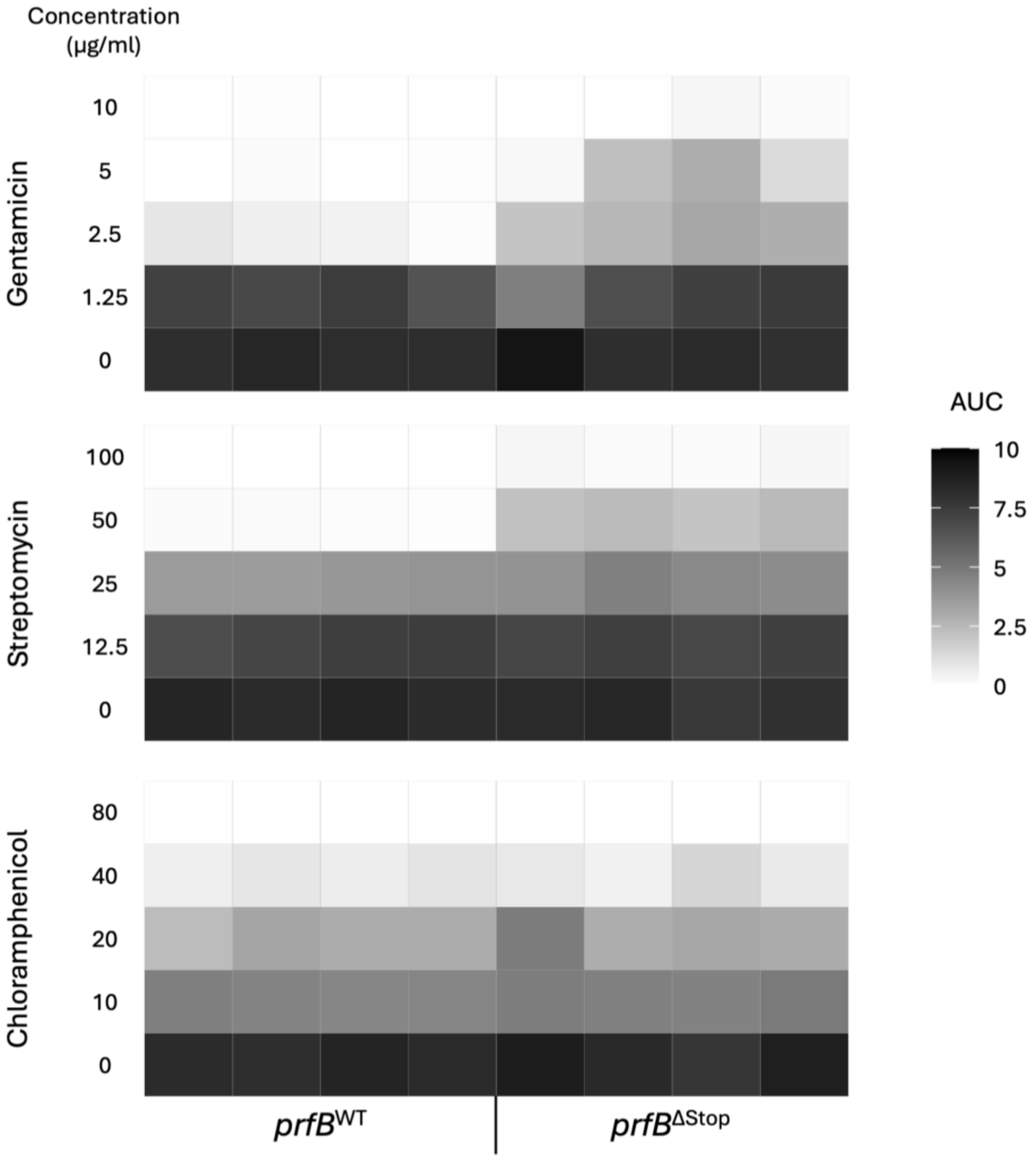

Supplementary Figure S4. Impact of deletion of autoregulation in *prfB* on antibiotic resistance. Area under the growth curve (AUC) during 24 hours of growth with the indicated *P. fluorescens* strains (bottom of each panel) in KB medium with different antibiotics at the indicated concentrations. Heatmaps of four replicates are shown per strain and antibiotic concentration.

Alt Text: Supplementary Figure S4 compares growth of wild-type *Pseudomonas fluorescens* SBW25 and a mutant lacking *prfB* autoregulation across a range of antibiotics and concentrations. Each panel shows growth in KB medium in the presence of a different antibiotic, with color intensity representing the amount of growth measured over 24 hours. Four replicate measurements are shown for each strain and condition. The figure allows comparison of how loss of *prfB* autoregulation affects antibiotic sensitivity.

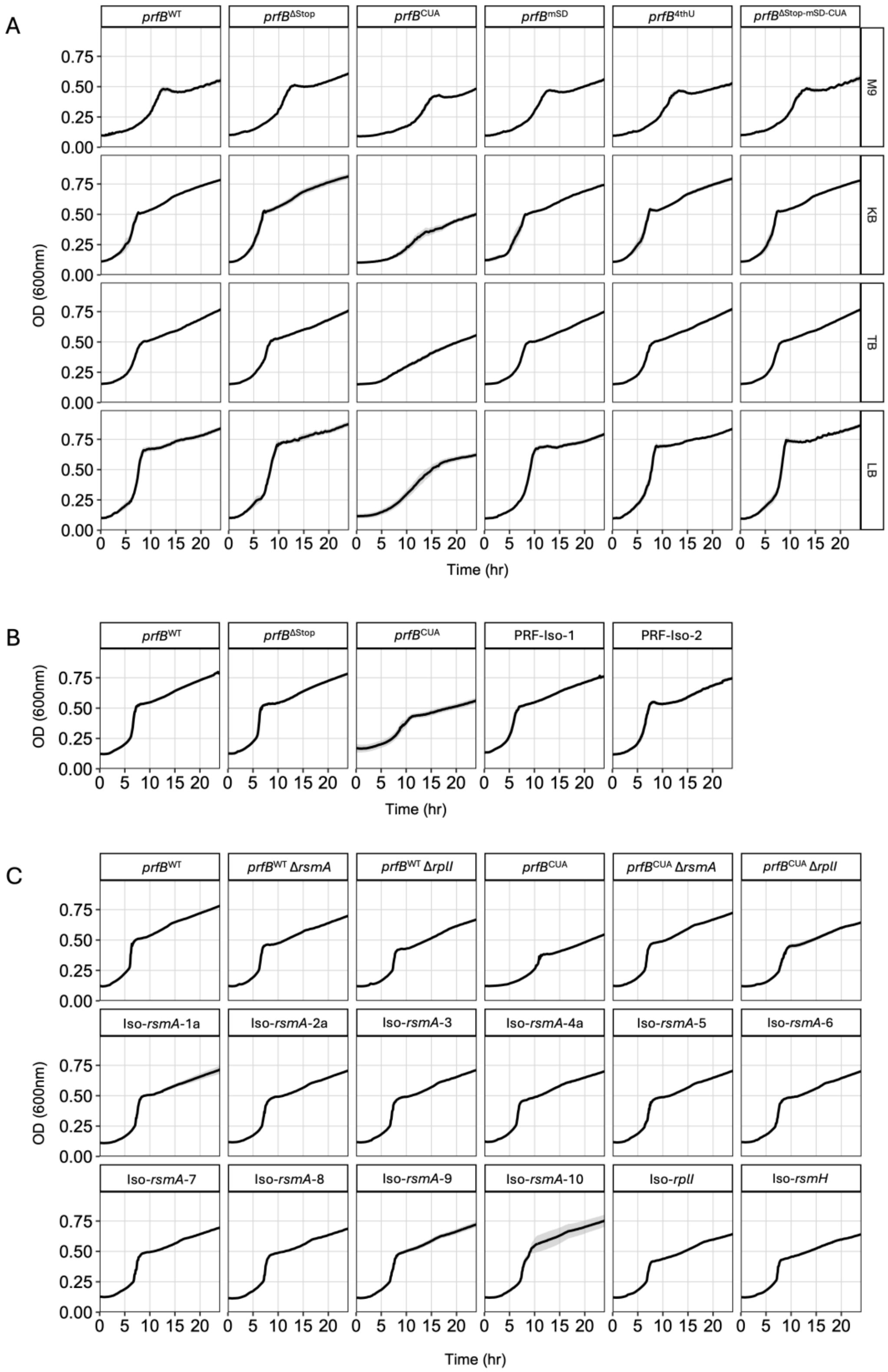
A
prfB^WT
prfB^ΔStop
prfB^CUA
prfB^mSD
prfB^4thU
prfB^ΔStop-mSD-CUA
M9
KB
TB
LB
OD (600nm)
Time (hr)
B
prfB^WT
prfB^ΔStop
prfB^CUA
PRF-Iso-1
PRF-Iso-2
OD (600nm)
Time (hr)
C
prfB^WT
prfB^WT ΔrsmA
prfB^WT ΔrplI
prfB^CUA
prfB^CUA ΔrsmA
prfB^CUA ΔrplI
Iso-rsmA-1a
Iso-rsmA-2a
Iso-rsmA-3
Iso-rsmA-4a
Iso-rsmA-5
Iso-rsmA-6
Iso-rsmA-7
Iso-rsmA-8
Iso-rsmA-9
Iso-rsmA-10
Iso-rplI
Iso-rsmH
OD (600nm)
Time (hr)

80

Supplementary Figure S5. Growth curves of the wild type, *prfB* mutants, and evolved isolates. Growth curves on a 96-well plate that are used to calculate the area under the curve (AUC) values are plotted in different main figures. KB medium was used unless specifically indicated. (**A**) Growth curves for Figure 3B. Each column indicates a different strain, and each row indicates a different growth medium. (**B**) Growth curves for Figure 4B. (**C**) Growth curves for Figure 4C. Details on the medium, replicates, and strains are provided in the captions of the main figures. The black line indicates the average OD (600nm), and the grey area indicates the standard error across replicates.

Alt Text: Supplementary Figure S5 shows the original growth curves used for the calculation of the area under the curve (AUC) shown in different main figures.

Supplementary Methods. Details about different experimental evolution runs

Three independent evolution experiments were performed (Exp1 to Exp3). The specific experimental setup for each experiment was as follows:

Exp1

- Lineage composition: 8 independent lineages of SBW25-*lacZ* and 8 independent lineages of the *prfB*^{CUA} mutant.
- Transfer volume: 4 lineages of each genotype were transferred with 4 µl (1:1000 dilution), and the other 4 lineages were transferred with 40 µl (1:100 dilution).
- Excluded lineages: One *prfB*^{CUA} lineage was excluded due to contamination.
- All isolates were selected at Week 1.

Exp2

- Lineage composition: 1 lineage of SBW25-*lacZ* and 16 independent lineages of the *prfB*^{CUA} mutant.
- Transfer volume: 40 µl for all lineages (1:100 dilution).
- Excluded lineages: One *prfB*^{CUA} lineage was excluded due to contamination.
- All isolates were selected at Week 1.

Exp3

- Lineage composition: 2 independent lineages of SBW25-*lacZ*, 16 independent lineages of the *prfB*^{CUA} mutant.
- Transfer volume: 40 µl for all lineages (1:100 dilution).
- Excluded lineages: Six *prfB*^{CUA} lineages were excluded due to contamination.
- In one lineage, a single isolate, Iso-rsmA-10, was isolated on Week 1, and another isolate, Iso-PRF-2, was isolated on Week 3. All other isolates were acquired separately from each lineages in Week 1.

The table below indicates the number of isolates obtained from the different experimental runs:

Gene/Allele ID	Exp1	Exp2	Exp3	Total
prfB / Iso-PRF-1	2			2
prfB / Iso-PRF-2	1		1	2
rsmA / Iso-*rsmA*-1a	1	1		2
rsmA / Iso-*rsmA*-1b			1	1
rsmA / Iso-*rsmA*-2a		1		1
rsmA / Iso-*rsmA*-2b	1			1
rsmA / Iso-*rsmA*-3		1		1
rsmA / Iso-*rsmA*-4a	1	9	4	14
rsmA / Iso-*rsmA*-4b			1	1
rsmA / Iso-*rsmA*-5	1			1
rsmA / Iso-*rsmA*-6		1		1
rsmA / Iso-*rsmA*-7		1		1
rsmA / Iso-*rsmA*-8			1	1
rsmA / Iso-*rsmA*-9			1	1
rsmA / Iso-*rsmA*-10			2	2
rsmH / Iso-*rsmH*		1		1
rplI / Iso-*rplI*		1		1
Total	7	16	11	34

Kim J, Na S-I, Kim D *et al.* UBCG2: Up-to-date bacterial core genes and pipeline for phylogenomic analysis. *J Microbiol* 2021;**59**:609–15.
